# Circularly polarized laser emission induced in isotropic and achiral dye systems

**DOI:** 10.1038/srep28740

**Published:** 2016-06-28

**Authors:** Luis Cerdán, Sara García-Moreno, Angel Costela, Inmaculada García-Moreno, Santiago de la Moya

**Affiliations:** 1 Instituto de Química Física “Rocasolano” (IQFR), Consejo Superior de Investigaciones Científicas (CSIC), C/Serrano 119, 28006, Madrid (Spain); 2 Departamento de Química Orgánica I, Facultad de Ciencias Químicas, Universidad Complutense de Madrid (UCM), Ciudad Universitaria S/N, 28040, Madrid (Spain)

## Abstract

The production of efficient, tunable, and switchable circularly polarized laser emission would have far reaching implications in optical communications or biophotonics. In this work, it is demonstrated the direct generation of circularly polarized (CP) laser emission in achiral and isotropic dye laser systems without the use of extracavity polarizing elements, and without resorting to chiral dyes, chiral liquid crystal matrices, or interferometric methods. The origin of this ellipticity arises from the dynamic birefringence induced by the strong and polarized laser pumping and the subsequent orientation anisotropy of the excited molecular dipoles. A complete polarimetric characterization of the polarization state of conventional dye laser oscillators as a function of different experimental parameters is performed and it is shown that the generated light always possesses a certain level of circularity that changes in a distinctive way with pump energy and polarization. These results demonstrate that it is possible to generate and modulate CP laser light from efficient and photostable conventional laser dyes.

Chirality, the asymmetry property by which an object or structure and its mirror image are not superimposable, plays an important role in material science^1^,^2^. Photonics is not an exception, and the growing interest on chiroptical phenomenon finds its roots in the resolution provided by the circular polarization (CP), which becomes essential in many advanced photonic technologies, such as information storage and processing^3^,^4^, communication of spin information^5^ or ellipsometry-based tomography^6^,^7^. Going one leap further, the direct generation of tunable and circularly polarized laser radiation is an essential step to promoting the implementation of chirality-based microscopy^8^,^9^,^10^ as an envisaged technology which would enable improved resolution in the omnipresent biological chiral environments.

Up to date the most common approaches for directly generating visible, tunable and circularly polarized laser radiation involve either the use of inherently chiral media, such as dye-doped cholesteric liquid crystals^11^,^12^,^13^,^14^,^15^, or the periodic spatial modulation of the refractive index of achiral media (dye-doped solution in liquid and solid phase) induced by interfering two circularly polarized laser beams from the output of the same laser^16^,^17^,^18^.

A more recent strategy focuses on the design and synthesis of chiral organic dyes enabling highly efficient, stable and tunable broad-line-width laser emission^19^,^20^. In fact, chiral organic laser dyes encompass a high technological potential, mainly related to the properties associated to their small size, and their excellent organic-solvent solubility. However, the development of chiral-laser organic dyes has been scarce because these systems have intrinsically two serious limitations: (1) require high enantiopurity, usually associated with entangled synthetic protocols that raise the cost of the emissive material, and (2) absolute luminescence dissymmetry factors |*g*_*lum*_| extremely small (typically between 10^−5^–10^−2^).

To develop CP laser technologies to its full potential, we designed a new strategy to laser-induced chirogenesis in achiral isotropic media with the aim to simultaneously overcome the limitations imposed by the synthesis of bespoke chiral dyes, and the complexity associated to engineered CP laser in achiral gain media by the spatial modulation of the index-grating induced by interfering two polarized pump beams. In this article, we report an unprecedented strategy to generate and modulate efficient and photostable CP laser emission from achiral laser dyes commercially available dissolved in achiral isotropic solvents and transversely pumped in a plane-parallel cavity without involving either laser beams interference or optical–chiral elements, in contrast to the previous approaches. We have performed a complete polarimetric characterization of the polarization state of conventional dye laser oscillators and identified the key factors governing the CP-laser generation, analyzing its dependence on experimental parameters such as the energy and polarization of the pump laser, the dye structure, the solvent viscosity or the cavity configuration. We unveil the experimental conditions under which achiral laser dyes enable highly efficient (70%) and circularly polarized laser emission, quantified by *g*_*lum*_ factors up to ~0.5, 100-fold higher that those registered up to now from small, non-polymeric and non-aggregated chiral organic dyes^19^,^20^, and approaching those exhibited by the weakly emissive lanthanide complexes^21^,^22^,^23^. Our present results demonstrate that the unique properties of laser radiation are sufficient to manipulate the physical behaviour in materials as to transform achiral organic dyes into an enantiopure-like system without altering its chemical identity. In this way, our strategy is a powerful approach to open new avenues for developing cost-effective and easy processable chiro-photonic elements and media.

## Polarimetry Formalism

The global polarization state of a given arbitrary beam can be described in terms of the Stokes parameters *S*_i_ (i = 0, 1, 2, 3)^24^, which are related to the beam energy (*E*_*out*_), the degree of polarization (*DOP*), and the orientation (*ψ*) and ellipticity (*χ*) of the polarization ellipse as:


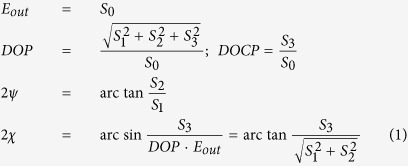


**Figure 1:**
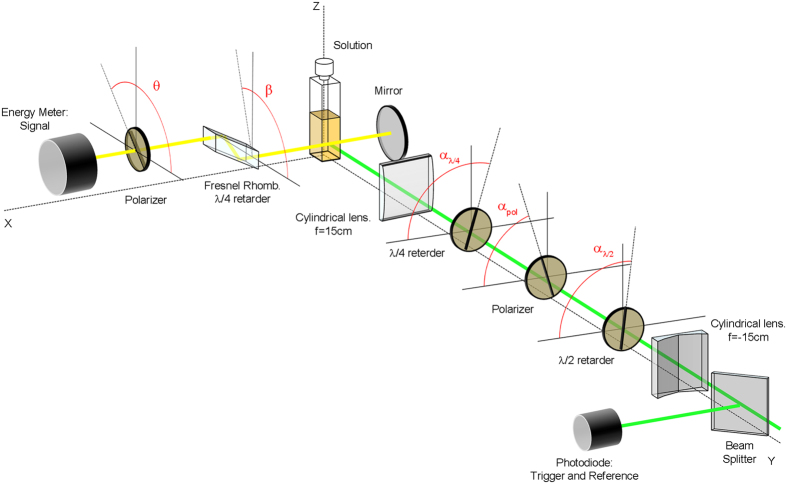
Experimental set-up for laser and polarimetry measurements: Sketch of experimental set-up used to determine the polarization state of a dye laser as a function of the pump polarization state (See Methods for description of elements). The optical elements axis angles and ellipticity direction of the different beams are determined looking towards the source of light.

For convenience, we have included in equation (1) the degree of circular polarization (*DOCP*), which will be used along the text. To measure the Stokes vector, we have used a home-made polarimeter with a rotating polarizer (or analyzer) and a static λ/4 retarder, a Fresnel rhomb in our case (see Fig. 1), and applying the Mueller matrix formalism^25^ (See Supplementary Notes for a complete description of the method). To retrieve *S*_*0*_ to *S*_*3*_ two sets of measurements must be performed. The first set corresponds to the energy of the beam after it has crossed the polarizer as a function of the polarizer transmission axis angle *θ*, which follows the expression:


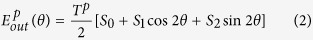


where *T*^*p*^ is the polarizer transmission. The second set corresponds to the energy of the beam after it has crossed first the λ/4 retarder with its fast axis at a given angle *β* (~π/2) and then the polarizer as a function of *θ*. In this case, the output energy will depend on *θ* as:


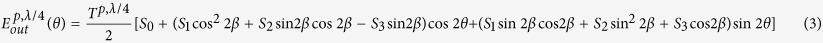


where *T*^*p*,*λ/4*^ is the polarizer/rhomb combined transmission. A simultaneous fit of equations (2) and (3) to the first and second sets of data, respectively, will provide *S*_*0*_ to *S*_*3*_ as fitting parameters. Once the Stokes parameters are known, the polarization state in terms of *E*_*out*_, *DOP*, *DOCP*, *ψ* and *χ*, can be calculated using equation (1). There are alternative parameters to account for the degree of circularization or ellipticity of a given light beam. For example, the works dealing with Circularly Polarized Luminescence^8^,^9^,^19^,^20^,^21^,^22^,^23^ (CPL) make use of the so-called dissymmetry factor *g*_*lum*_, which is minus twice the degree of circular polarization (*DOCP*) as defined in equation (1). *g*_*lum*_ will be as well evaluated in our work.

## Experimental Results

Once the polarimeter was adjusted and calibrated (see Methods), we proceeded to evaluate the dye laser properties (efficiency and polarization state) as a function of pump polarization and energy. Four different and representative pump polarizations were used throughout the paper, namely, linear vertical or perpendicular to the cavity axis (↕), linear 45° over the horizontal (↗), linear horizontal or parallel to the cavity axis (↔), and left handed (↺) circular. We observed that the handedness of the circularly polarized pump did not affect the output polarization state. Consequently, we will focus through the rest of the paper only on the left handed (↺) circular polarization. Something analogous happens with the pump linearly polarized at 135° and 45°. As lasing medium we chose a dissolution of the achiral laser dye PM567 at a concentration 5×10^−4^ M with ethyl acetate (EtOAc) as solvent.

**Figure 2:**
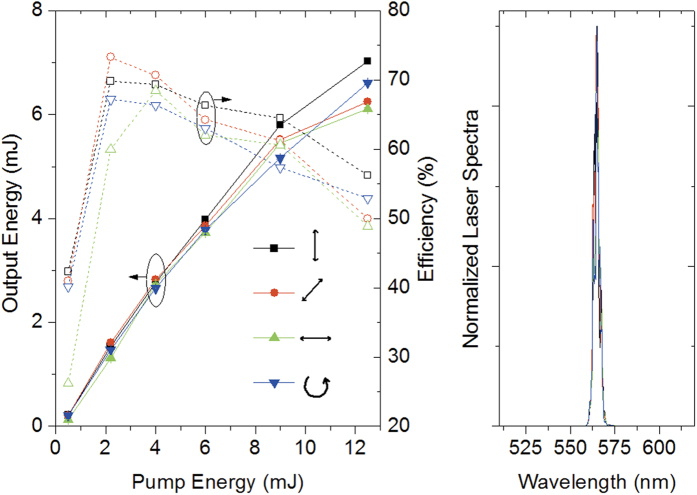
Laser performance of PM567 5 × 10^−4^ M in EtOAc: (**a**) output energy (solid symbols) and efficiency (open symbols) as a function of pump energy and polarization. (**b**) Laser spectra as a function of pump polarization.

Figure 2 shows the output laser energy and efficiency (ratio of output and input energies) and the laser spectra as a function of both pump polarization and energy. Regarding the pump polarization effects, the best laser performance is obtained pumping with a vertical polarization, but not by far. In fact, as was previously reported^26^, the efficiency and the laser spectra, peaked at 565 nm (Fig. 2), are quite insensitive to the pump polarization state, at least for our cavity configuration and sample.

**Figure 3:**
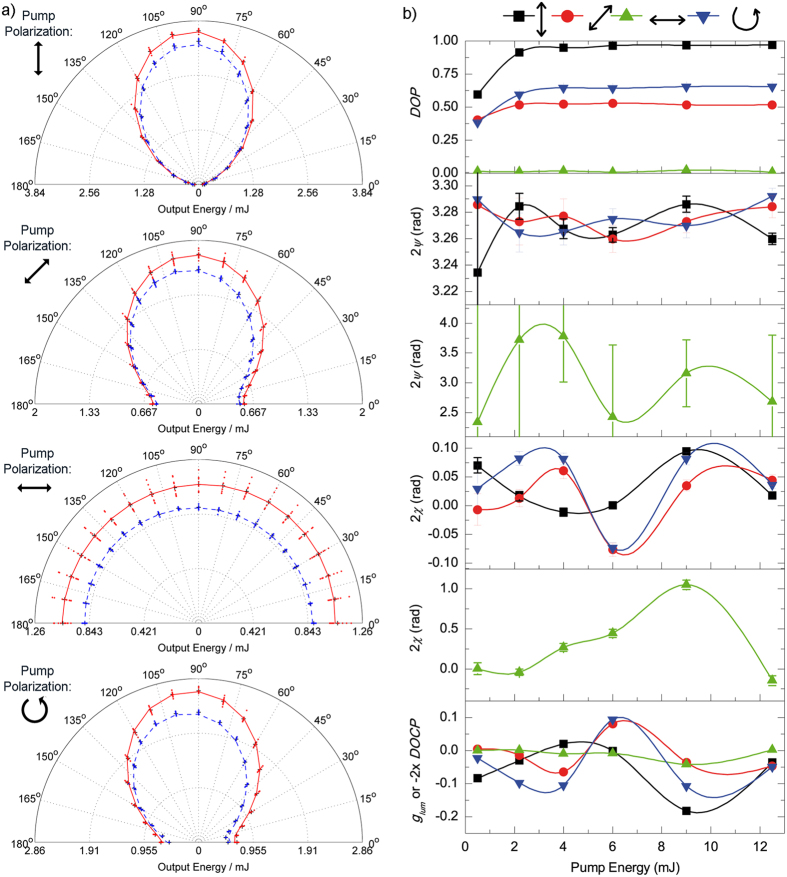
Pump energy and polarization influence on dye laser polarization state: (**a**) Output energy as a function of the analyzer transmission axis angle *θ* without (red data) and with (blue data) inserting a λ/4 retarder in the beam path (*E*_*pump*_ ~ 4 mJ). Dots are experimental measurements and lines are best fits of equations (2) and (3). (**b**) Laser emission polarization state parameters for different pump energies and polarizations. Dots are fitting parameters and lines are guides to the eye. *2ψ* and *2χ* parameters corresponding to the horizontally polarized pump (▴) are plotted separately due to dissimilar scales. Sample: PM567 5×10^−4^ M in EtOAc. Each point and error bar in (**b**) represents the weighted average and error of the fitting parameters obtained over 10 sets of measurements.

All the other polarization state parameters (*DOP*, *DOCP*, *2ψ*, *2χ*, and *g*_*lum*_) are indeed sensitive to pump polarization. Figure 3a shows a particular example of the polar graphs obtained for each of the pump polarizations at a given pump energy. The most evident change in these graphs concerns the *DOP*, which follows the expected behaviour^27^,^28^ going from almost unity (totally polarized), when the pump polarization is vertical, to almost zero (fully depolarized) when the pump polarization is horizontal, and rendering a partial polarization for both linear 45° and left-handed circular pumping (see Fig. 3b).

At this point, it must be noted that most experimental works on conventional dye lasers only account for the intensity and *DOP* of the output emission^28^,^29^,^30^,^31^, and these define the *DOP* as (*I*_*max*_ − *I*_*min*_)/(*I*_*max*_ + *I*_*min*_) where *I*_*max*_ and *I*_*min*_ are the radiation intensities recorded through the analyzer (in the absence of a λ/4 retarder) at the two mutually orthogonal positions corresponding to the maximum and minimum output intensities. This expression would render a *DOP* = 0 for fully circularly polarized light, being a paradox and a source of errors in data interpretation, revealing the need of using an adequate polarimeter to study the polarization state of lasers.

Coming back to the results on Fig. 3, the *DOP* depends on both pump polarization and pump energy as it is well-known^26^,^27^,^28^,^29^,^30^,^31^,^32^. Whereas the trend with the pump polarization state is always the same irrespective of the pump energy (*DOP*_↕_ > *DOP*_↺_ > *DOP*_↗_ > *DOP*_↔_), at low pump energies the emission is consistently less polarized than above saturation (~3 mJ), when the *DOP* stops increasing, in agreement with previous theoretical and experimental works^31^,^32^. This is related to the molecular rotation that takes place upon excitation, as will be detailed in the discussion section.

Figure 3 also shows that pump energy and polarization have an effect on the behaviour of the rest of polarization state parameters. It can be seen that the polarization state is oriented around the vertical (*2ψ* ~ 3.27), except for horizontally polarized pump, in which case orientation is difficult, if not meaningless, to define, with errors much larger than the values themselves. The vertical orientation of the emission polarization is also well-known^27^, and finds an explanation similar to the reasoning about the *DOP*. Nevertheless, for every polarization, the value of *2ψ* depicts a non-trivial non-periodic oscillation as the pump energy increases (Fig. 3b). We are not aware of any previous work reporting this behaviour.

The most striking and novel result of this paper is the change in the ellipticity of the polarized light with the pump energy and polarization. The visual inspection of the polar plots in Fig. 3a not always distinctively reveals the presence of elliptically polarized light, whereupon is better to resort to the values of *2χ* obtained from the fitting procedure. In this regard, it is clear from our results (Fig. 3b) that, far from being zero (as would happen with linearly polarized light), *2χ* oscillates from positive (right-handed) to negative (left-handed) and back depending on the pump energy. In addition, the oscillation behaviour and levels of *2χ* depend on the pump polarization state. For example, for the vertical pump polarization those values range from a mere −0.01 rads to almost 0.1 rads; *i.e*., it presents a modest level of ellipticity. As a reminder, *2χ* can range from π/2 for right-handed circularly polarized light to −π/2 for the left-handed one. Significantly, when the pump is horizontally polarized, the output emission may reach a remarkable *2χ* value of 1 rad, *i.e*., almost circularly polarized. But care must be exercised with these values, as it does not suggest that the polarization state of the dye laser emission is highly elliptical, it only informs that the part of the light that is polarized (*i.e*., the *DOP*) is elliptical. For example, in the case of horizontal pumping, no matter how circular the polarized contribution is, the output laser light will be virtually depolarized (*DOP* ~ 0.01). To account for the total ellipticity of the output emission one should resort to the *DOCP* or *g*_*lum*_. Using again as an example the previous case, the output laser light would have, in the best case, a *DOCP* ~ 0.02 (*g*_*lum*_ ~ −0.04), *i.e*., just a 2% of elliptical polarization. On the contrary, the laser light generated by the vertical pump polarization, with a value of *2χ* as modest as 0.1 rads, renders a *DOCP* ~ 0.09 (*g*_*lum*_ ~ −0.18), *i.e*., it has a 9% global ellipticity. It is remarkable that the output light with the highest *DOCP* it is obtained with the highest levels of *DOP* (vertical pump polarization).

In order to check that the observed output light ellipticity was not due to an unknown intrinsic property of the achiral PM567, analogous experiments were conducted with two other dyes, Rhodamine 6G (Rh6G) and Pyridin 1 (or LDS698), both dissolved in ethanol. Rh6G is photophysically analogous to PM567 (narrow bands, small Stokes shift, nanosecond fluorescence lifetime, parallel absorption and emission dipole moments, and absorption and emission taking place from the same spatial location), whereas LDS698 - intramolecular charge transfer dye^33^– is photophysically dissimilar to both PM567 and Rh6G (broader bands, huge Stokes shift, subnanosecond lifetimes, non-parallel absorption and emission dipole moments, and absorption and emission taking place from different spatial location). As seen in Table 1, the laser emission registered from all the selected dyes always presents a given level of ellipticity that changes with the pump polarization state. These results pinpoint to a non-inherent property of the dyes as responsible for the CP-laser light generation.

## Discussion

It is widely recognized that the intense laser pump radiation in dye laser systems induces an anisotropy in the solution that affects the polarization state of the amplified laser radiation^34^,^35^,^36^,^37^,^38^. This anisotropy is induced by two different effects, a phase anisotropy due to a difference in refractive indices “feel” by the different cavity modes, and an amplitude anisotropy due to a dissimilar amplification of them. Most experimental works thoroughly studying the laser induced anisotropy have been carried out in amplifiers^34^,^35^,^36^,^37^,^38^. On the other hand, the existing experimental works dealing with the oscillator only focused on the output intensity and the degree of polarization^26^,^28^,^29^,^30^,^31^,^32^, and the complete picture has only been dealt theoretically^35^, with no quantitative results available, hence the relevance of the present work.

### Degree and orientation of polarization (DOP and 2ψ)

**Figure 4:**
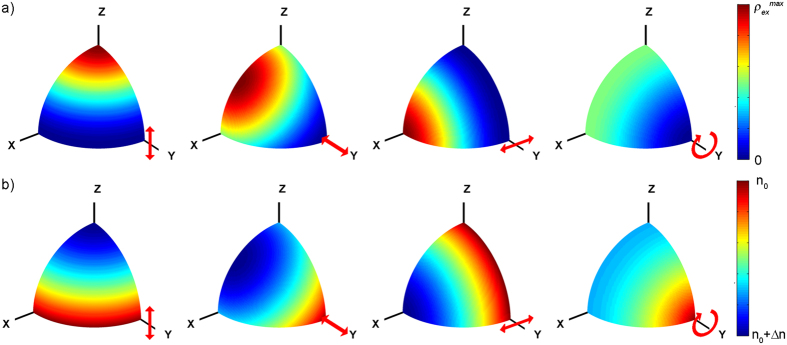
Orientation of excited state population and refractive index ellipse for each pump polarization: Qualitative representation of the orientation dependant density of excited state molecules (**a**) and refractive index (**b**) for different pump polarizations (before thermalization and molecular rotation). The coordinate labels are coincidental with those in Fig. 1. The pump beam and cavity axis are parallel to the Y- and X- axis, respectively. The red arrows show the pump electric field orientation. See Supplementary notes for a detailed description on the formulation behind these plots.

The observed trend of *DOP*, which is fully consistent with previous reports^27^,^28^, can be unambiguously explained attending to the orientation anisotropy of the excited molecular dipoles upon pumping (Fig. 4a). Only the molecules whose absorption dipole moments unit vectors **e**_**d**_ are properly aligned with the electric field **E**_**p**_ of the pump radiation will be excited. The probability of being excited will be then proportional to |**E**_**p**_**·e**_**d**_|^2^, *i.e*., it will be proportional to cos^2^ϑ, where ϑ is the dihedral angle formed by the absorption dipole moment and the electric field vector (see Supplementary Notes for a detailed description). Once the molecules have been excited, they must rotate so that their emission dipole moment is coupled to any of the cavity modes, *i.e*., perpendicular to the resonator axis and lying in the ZY plane. For example, when the pump polarization is linear vertical (along the Z-axis), most excited molecules already lie perpendicular to the cavity axis (Fig. 4a) and can emit before rotation relaxation is effective. As they are mainly vertically oriented, most of the emission will be vertically polarized, rendering a *DOP* reaching unity with a value for *2ψ* close to π, as can be seen in Fig. 3b. On the contrary, when the pump polarization is linear horizontal (along the X-axis), not only none of the dipole moments lie in the ZY plane, but they are mostly perpendicular to it (Fig. 4a), and hence they must rotate to couple to a cavity mode. After rotation, the probability of being aligned with the Z-axis and the Y-axis is the same, and hence the intensity in any of these two modes will be the same. In other words, the emission will be mostly depolarized (*DOP* ~ 0) and the orientation of the polarization ellipse will be difficult, if not meaningless, to define. Pumping with linear polarization 45° and circular polarization are intermediate cases (Fig. 4a), giving values of *DOP* ~ 0.5–0.6, and orientations *2ψ* close to π, as the rotation towards the Z-axis is shorter than that towards the Y-axis. Of course the previous reasoning is only valid provided the absorption and emission dipole moments are parallel^27^.

The increase of *DOP* with the pump energy is related to the molecular rotation that takes place upon excitation. If the rotational relaxation rate *γ*_*rot*_ (inverse of time the molecule needs to randomize its orientation) is larger than the emission rate *γ*_*em*_ = *σ*_*st*_·*I*_*L*_/(*hν*_*l*_) (*σ*_*st*_ is the stimulated emission cross section, *I*_*L*_ is the intracavity laser intensity, *h* is the Planck constant, and *ν*_*l*_ is the laser frequency), the molecules will have time to rotate before emitting, thus randomizing the dipole moment orientation and reducing, consequently, the *DOP*. When *γ*_*rot*_ ≪ *γ*_*em*_ the opposite is true, and the molecules are “effectively” frozen. Hence, for low pump energies *γ*_*rot*_ > *γ*_*em*_, and the emission tends to be less polarized, as is clearly shown in Fig. 3b. As the pump intensity grows the laser intensity increases (see Fig. 2) and, consequently, so does *γ*_*em*_, eventually becoming larger than *γ*_*rot*_ at pump energies above 3 mJ. Small molecular systems in liquid solvents (such as PM567 in EtOAc) depict a rotation time of tens to hundreds of picoseconds^39^. The use of solvents in which the rotation time takes significantly longer, like more viscous solvents or polymer matrices, allows reaching *γ*_*rot*_ rate smaller than *γ*_*em*_ irrespective of the pump energy. Accordingly, PM567 (5×10^−4^ M) in a mixture of EtOAc and PMMA at a wt/wt proportion 40:60 -enough to render a viscous solution without modifying the photophysical properties of the dye- exhibits, irrespective of the pump polarization, *DOP* slightly higher than in neat EtOAc (Table 1).

### Ellipticity of polarization (2χ)

The oscillation behaviour observed for 2*ψ* and 2*χ* finds an explanation again in the orientation anisotropy of the excited molecular dipoles. As explained before, the pump radiation excites preferentially molecular dipoles that are aligned with its electric field vector (Fig. 4a). Consequently, the molecules remaining in the ground state will present an orientation anisotropy that is conjugated with respect to that of the excited molecules. This, in turn, renders an absorption orientation anisotropy. Since the real and imaginary parts of the medium susceptibility are connected by a causality relationship (Kramers-Kronig relations, see Supplementary Notes), the presence of an absorption orientation anisotropy will causally translate into a refractive index orientation anisotropy, *i.e*., an optical birefringence is induced. This birefringence is responsible for the phase anisotropy, in which each cavity mode travels at a different speed within the medium, giving place to a given phase delay between them.

As can be appreciated in the qualitative representation of Fig. 4b, the transversal pumping (parallel to the Y-axis) gives place to a very distinctive refractive index distribution. Irrespective of the particular pump polarization, the active medium becomes a sort of uniaxial “crystal” in which the refractive index of one axis (extraordinary) is different from that of the other two ordinary axes (*i.e. n*_*i*_ ≠ *n*_*j*_ = *n*_*k*_). Whereas the three linear pump polarizations generate negative uniaxial media (*n*_*e*_ < *n*_*o*_), the circularly polarized pump generates a positive uniaxial medium (*n*_*e*_ > *n*_*o*_). Nevertheless, as the laser light travels along the cavity optical axis, it “feels” a negative uniaxial medium with the fast axis (that with the lowest refractive index) parallel to the Z-axis irrespective of the pump polarization. Hence, the effective fast axis orientation is always perpendicular to both the incident direction of the pump beam and the cavity axis.

Once the birefringence Δ*n* has been set up in the dye solution, it behaves as an effective linear retarder with retardance Γ and fast axis oriented a given angle *θ*_*L*_. Hence, any partially polarized light propagating in it will see its polarization state modified^40^. Unlike chiral dyes, that emit elliptically polarized radiation, common dyes such as the ones used in these experiments emit photons that are linearly polarized. Hence, the light that is generated and amplified in the laser cavity is linear, but it acquires a certain level of ellipticity as it travels through the dye solution, explaining the values of 2*χ* measured with our polarimeter (Fig. 3). Of course, the real picture is much more complex, as the molecules randomize their orientation through thermalization and rotational-relaxation, thus modifying the strength of the anisotropy with time. On top of this, the density of population in the excited-state, and in turn the refractive index variation, depends on the pump energy, position within the solution, and time^35^. This complex and multiparametric dependant refractive index anisotropy may explain the surprising oscillation behaviour shown by 2*ψ* and 2*χ* (Fig. 3).

If the dye solution behaves as an effective linear retarder with retardance Γ, an inversion of the beam propagation direction would generate light with an opposite circularity. To check this fact, we repeated the experiment leaving the pump geometry unchanged, but moving the mirror (and the polarimeter) to the other edge of the cavity (see Supplementary Fig. S4). As summarized in Table 2, with this “inverse cavity” the *DOP* is unchanged, whereas the value of 2*χ* (and of *g*_*lum*_) is opposite to that obtained with the “normal cavity”, confirming the above reasoning.

Finally, to check that the effective linear retarder fast axis orientation angle *θ*_*L*_ is determined by the pump incidence angle, we repeated the experiment leaving the pump geometry and the cavity configuration unchanged, but tilting the cuvette towards the pump and away of the pump (see Supplementary Fig. S4). The *DOP* and 2*χ* (and *g*_*lum*_) are not modified by this tilting (Table 2), and only the polarization orientation 2*ψ* is slightly modified. This modification comes from the beam deviation exerted by the cuvette walls due to refraction (see Supplementary Fig. S5). This last experiment confirms that the fast axis orientation is determined by the normal of the pump beam incidence direction.

## Conclusion

In summary, we have performed a complete polarimetric characterization of the polarization state of conventional dye laser oscillators as a function of different experimental parameters (pump energy and polarization, active medium viscosity, cuvette orientation). We have shown that the generated light always possesses a certain level of circularity that changes in a distinctive way with pump energy and polarization. This reveals the need of using an adequate polarimeter to study the polarization properties of dye lasers. The origin of this ellipticity arises from the dynamic birefringence induced by the strong and polarized laser pumping and the subsequent orientation anisotropy of the excited molecular dipoles. These results demonstrate that it is possible to generate and modulate CP laser light from efficient and photostable conventional laser dyes without the use of extracavity polarizing elements, and without resorting to chiral dyes, chiral liquid crystal matrices, or interferometric methods. In conclusion, our strategy opens new avenues for developing cost-effective and easily processable chiro-photonic elements and media, in particular for optofluidic lasers^41^.

## Methods

### Materials

Dyes PM567, Rhodamine 6G and LDS 698 were laser grade and purchased from Exciton. Solvents from laser studies were spectroscopic grade (Merckel, Aldrich or Sigma) and were used without purification. Poly(methyl methacrylate) (PMMA) of 25,000 molecular weight (Polysciences, Inc.) was used as purchased.

### Experimental set-up

A sketch of the experimental set-up used in this work is depicted in Fig. 1. The dyes dissolved in organic solvents were placed in 1 cm optical path quartz cuvettes and optically pumped at 532 nm with a frequency-doubled Q-switched Nd:YAG laser (Lotis TII SL-2132) emitting pulses 20 ns full width at half maximum (FWHM) and operated at 15 Hz repetition rate. Pump energy was measured with a calibrated pyroelectric energy meter (ED200, GenTec). The pump laser radiation was horizontally polarized, which allows controlling the pulse energy incident on the sample by insertion into the pump beam path of a half-wave plate (HWP) and a linear polarized (LP) set with its polarization axis horizontal or vertical, depending on the desired final pump polarization. By rotating the HWP, the linear polarization of the input beam is rotated out of the horizontal, and the pump beam is blocked more or less by the LP, depending on the rotation angle introduced by the HWP. To obtain a circularly polarized pump beam a quarter-wave plate (λ/4 retarder) was placed after the LP. In the transversal pumping configuration measurements, the light incident on the sample was perpendicularly to the surface of the cuvette and focused onto that surface in a stripe shape of ∼ 300 μm width by a combination of negative (NCL) and positive (PCL) cylindrical quartz lenses (f = −15 and +15 cm, respectively) perpendicularly arranged. The oscillation cavity (2 cm length) consisted of a 90% reflectivity aluminium back mirror and the end face of the cuvette as output coupler. A beam splitter was used to send a reflection of the pump beam into a photo-diode acting as the trigger. The trigger signal was fed to a boxcar (Standford Research, model 250) to convert it to a delayed TTL pulse to trigger the digital oscilloscope (Yokogawa, model DL1620). The photo-diode signal was also sent to the oscilloscope and used as reference signal to monitor and control the pump energy along the experiments. To analyze the polarization degree and state of the dye laser emission we tailored a polarimeter based on a Fresnel rhomb, acting as a quarter-wave plate, combined to a linear polarized (Thorlabs LPVISB100-MP) and a pyroelectric energy meter, which signal was registered with the oscilloscope.

### Polarimetry measurement protocol and calibration

A stringent measurement protocol was devised to guarantee a good accuracy on measuring 2*χ*. A single energy measurement (each point in Fig. 3a) was averaged over 32 pulses. For each analyzer angle *θ*, 10 energy measurements were acquired. This protocol enables an accuracy of ±5 mrad. To calibrate the Fresnel rhomb fast axis orientation *β*, we placed a linear polarizer between the dye laser cavity and the polarimeter, and fitted equations (2) and (3) (with *S*_3_ set to zero and leaving *S*_*0*_, *S*_*1*_, *S*_*2*_, and *β* free) to the sets of data without and with λ/4 retarder, respectively. This procedure renders a fitted value of *β* ~ 93.76° ± 0.01. See Supplementary Notes for a more detailed description.

## Additional Information

**How to cite this article**: Cerdán, L. *et al*. Circularly polarized laser emission induced in isotropic and achiral dye systems. *Sci. Rep*. **6**, 28740; doi: 10.1038/srep28740 (2016).

## Supplementary Material

Supplementary Information

## Figures and Tables

**Table 1:** Polarization state parameters of laser emission from different active media. *E*_*pump*_ ~ 4.4 mJ; Dye concentration: 5×10^−4^ M.

Pump Polarization	*E_out_* (mJ)	*DOP*	2*ψ* (rad)	2*χ* (rad)	*g_lum_* (=−2 × *DOCP*)	Sample
↕	2.77	0.95	3.27	−0.011. ± 0.001	0.02	PM567 in EtOAc
↗	2.83	0.52	3.28	0.061 ± 0.001	−0.06	PM567 in EtOAc
↔	2.74	0.01	3.78	0.27 ± 0.05	−0.01	PM567 in EtOAc
↺	2.66	0.64	3.26	0.081 ± 0.002	−0.10	PM567 in EtOAc
↕	2.59	0.99	3.28	0.033 ± 0.001	−0.06	Rh6G in ethanol
↗	2.72	0.54	3.25	0.057 ± 0.001	−0.06	Rh6G in ethanol
↔	2.62	0.008	3.74	−0.480 ± 0.05	0.007	Rh6G in ethanol
↺	2.53	0.70	3.26	0.049 ± 0.001	−0.07	Rh6G in ethanol
↕	1.58	0.98	3.28	−0.010 ± 0.001	0.02	LDS698 in ethanol
↗	1.39	0.71	3.29	0.340 ± 0.001	−0.47	LDS698 in ethanol
↔	1.13	0.007	2.71	−0.07 ± 0.09	0.001	LDS698 in ethanol
↺	1.36	0.85	3.28	0.230 ± 0.001	−0.39	LDS698 in ethanol
↕	2.69	0.98	3.27	0.112 ± 0.001	−0.22	PM567 in EtOAc:PMMA (40:60)
↗	2.51	0.57	3.26	0.110 ± 0.001	−0.13	PM567 in EtOAc:PMMA (40:60)
↔	2.42	0.02	3.31	1.02 ± 0.07	−0.03	PM567 in EtOAc:PMMA (40:60)
↺	2.56	0.75	3.26	0.113 ± 0.002	−0.17	PM567 in EtOAc:PMMA (40:60)

**Table 2:** Polarization state parameters of laser emission from PM567 5×10^−4^ M in EtOAc for different cavity configurations (*E*_*pump*_ ~ 2–3mJ).

Pump Polarization	*E*_*out*_ (mJ)	*DOP*	2*ψ*(rad)	2*χ* (rad)	*g*_*lum*_ (=−2 × *DOCP*)	Cavity type
↕	2.77	0.94	3.27	−0.010 ± 0.001	0.02	Normal cavity
↕	2.57	0.94	3.29	0.04 ± 0.001	−0.07	Inverse cavity
↗	3.28	0.50	3.24	0.037 ± 0.001	−0.04	Normal cavity
↗	3.36	0.52	3.28	−0.045 ± 0.001	0.05	Inverse cavity
↕	2.22	0.95	3.23	0.023 ± 0.001	−0.04	Cuvette tilted −5° pump-wards
↕	2.36	0.96	3.28	0.022 ± 0.001	−0.04	Cuvette tilted 5° pump-wards

## References

[b1] Wang Y., Xu J., Wang Y. & Chen H. Emerging chirality in nanoscience. Chem. Soc. Rev. 42, 2930–2962 (2013). 23207678 10.1039/c2cs35332f

[b2] Green D. W., Lee J.-M., Kim E.-J., Lee D.-J. & Jung H.-S. Chiral biomaterials: from molecular design to regenerative medicine. Adv. Mater. Interfaces, doi: 10.1002/admi.201500411

[b3] Wagenknecht C. . Experimental demonstration of a heralded entanglement source”. Nat. Photonics 4, 549–552 (2010).

[b4] Sherson J. F. . Quantum teleportation between light and matter. Nature 443, 557–560 (2006). 17024089 10.1038/nature05136

[b5] Farshchi R., Ramsteiner M., Herfort J., Tahraoui A. & Grahn H. T. Optical communication of spin information between light emitting diodes. Appl. Phys. Lett. 98, 162508 (2011).

[b6] Jan C. M. Integrating fault tolerance algorithm and circularly polarized ellipsometer for point-of care applications. Opt. Express 19, 5431–5441 (2011). 21445182 10.1364/OE.19.005431

[b7] Yu C. J., Lin C. E., Yu L. P. & Chou C. Paired circularly polarized heterodyne ellipsometer. Appl. Opt. 48, 758–764 (2009). 19183605 10.1364/ao.48.000758

[b8] Muller G. Luminescent chiral lanthanide(III) complexes as potential molecular probes. Dalton Trans. 9692–9707 (2009). 19885510 10.1039/b909430j PMC2784929

[b9] Seitz M., Moore E. G., Ingram A. J., Muller G. & Raymond K. N. Enantiopure, octadentate ligands as sensitizers for europium and terbium circularly polarized luminescence in aqueous solution. J. Am. Chem. Soc. 129, 15468–15470 (2007). 18031042 10.1021/ja076005e PMC2636552

[b10] Tsumatori H., Harada T., Yuasa J., Hasegawa Y. & Kawai T. Circularly polarized light from chiral lanthanide(III) complexes in single crystals. Appl. Phys. Express 4, 011601 (2011).

[b11] Furumi S. Recent progress in chiral photonic band-gap liquid crystals for laser applications. The Chemical Record 10, 394–408 (2010). 20954194 10.1002/tcr.201000013

[b12] Inoue Y. . Tunable lasing from a cholesteric liquid crystal film embedded with a liquid crystal nanopore network. Adv. Mater. 23, 5498–5501 (2011). 22081506 10.1002/adma.201102764

[b13] Karpinski P. & Miniewicz A. Optical phase conjugation in azo-dye doped chiral liquid crystal. Appl. Phys. Lett. 101, 161108 (2012).

[b14] Zheng Z.-G., Wang Ch. & Shen D. Dichroic-dye-doped polymer stabilized optically isotropic chiral liquid crystals. J. Mater. Chem. C 1, 6471–6478 (2013).

[b15] Li L.-W. & Deng L.-G. Random lasing from dye-doped chiral nematic liquid crystals in oriented and non-oriented cells. Eur. Phys. J. B 86, 112 (2013).

[b16] Lo D., Ye C. & Wang J. Distributed feedback laser action by polarization modulation. Appl. Phys B 76, 649–653 (2003).

[b17] Ye C., Wang J., Shi L. & Lo D. Polarization and threshold energy variation of distributed feedback lasing of oxazine dye in zirconia waveguides and in solutions. Appl. Phys. B 78, 189–194 (2004).

[b18] Chen F., Gindre D. & Nunzi J. M. Tunable circularly polarized lasing emission in reflection distributed feedback dye lasers. Opt. Express 16, 16746–16753 (2008). 18852784 10.1364/oe.16.016746

[b19] Sanchez-Carnerero E. M. . Circularly polarized luminescence by visible-light absorption in a chiral O-BODIPY dye: unprecedented design of CPL organic molecules from achiral chromophores. J. Am. Chem. Soc. 136, 3346–3349 (2014). 24524257 10.1021/ja412294s PMC3984031

[b20] Sanchez-Carnerero E. M. . Circularly polarized luminescent from simple organic molecules. Chem. Eur. J. 21, 13488–13500 (2015). 26136234 10.1002/chem.201501178 PMC4567477

[b21] Muller G. In Luminescence of Lanthanide Ions in Coordination Compounds and Nanomaterials (Ed.: de Bettencourt-Dias A.) 77–124 (Wiley, 2014).

[b22] Zinna F. & Di Bari L. Lanthanide circularly polarized luminescence: bases and applications. Chirality 27, 1–13 (2015). 25318867 10.1002/chir.22382

[b23] Heffern M. C., Matosziuk L. M. & Meade T. J. Lanthanide probes for bioresponsive imaging. Chem. Rev. 114, 4496–4539 (2014). 24328202 10.1021/cr400477t PMC3999228

[b24] Born M. & Wolf E. (eds) Principles of Optics, 5^th^ Ed. (Pergamon Press, 1975).

[b25] Chipman R. A. In Handbook of Optics II, 2^nd^ Ed. (ed. Bass M.) chapter 22 (McGraw-Hill, 1995).

[b26] Mikhailov I. V. Influence of the pump radiation polarisation on the efficiency of pulsed dye lasers. Quantum Electronics 25, 1176–1177 (1995).

[b27] Schäfer F. P. (ed.) Dye Lasers (Springer-Verlag, 1990).

[b28] Gancherenok I. I. & Shapochkina I. V. Degree of polarization of radiation of laser-pumped dye lasers with an isotropic resonator. Optics and Spectroscopy 80, 135–139 (1996).

[b29] Burakov V. S., Vasilev N. N., Gorolenko A. Ya., Sivenkova V. E. & Shkadarevich A. P. Study of the polarization characteristics of the radiation from a laser with dye-activated polymer elements. Zh. Prikl. Spektrosk. 42, 35–40 (1985).

[b30] Gancherenok I. I. & Shapochkina I. V. Polarization and energy parameters of isotropic dye lasers in the case of saturation in the orientation distribution of excited molecules. Optics and Spectroscopy 84, 109–115 (1998).

[b31] Kuznetsova R. T., Shaposhnikov A. A., Filinov D. N., Kopylova T. N. & Tel’minov E. N. Polarization characteristics of stimulated emission of organic molecules when excited by intense XeCl laser radiation. Optics and Spectroscopy 95, 447–454 (2003).

[b32] Gaisenok V. A., Gruzinskii V. V. & Krylov G. G. Effects of pump geometry and mutual orientation of absorption and emission dipoles on stimulated emission characteristics of dye solutions. Zh. Prikl. Spektrosk. 49, 26–31 (1988).

[b33] Cerdán L., Costela A., García-Moreno I., Bañuelos J. & López-Arbeloa I. Singular laser behavior of hemicyanine dyes: unsurpassed efficiency and finely structure spectrum in the near-IR región. Laser Phys. Lett. 9, 426–433 (2012).

[b34] Waldeck D., Cross A. J., McDonald D. B. & Fleming G. R. Picosecond pulse induced transient molecular birefringence and dichroism. J. Chem. Phys. 74, 3381–3387 (1981).

[b35] Haas R. A. & Rotter M. D. Theory of pulsed dye lasers including dye-molecule rotational relaxation. Phys. Rev. A 43, 1573–1603 (1991). 9905186 10.1103/physreva.43.1573

[b36] Chernyavsky V. A., Pikulik L. G., Rudik K. I. & Grib A. F. Polarization of radiation amplified by dye solutions. Quantum Semiclass. Opt. 10, 459–468 (1998).

[b37] Chernyavsky V. A., Pikulik L. G., Rudik K. I. & Grib A. F. Dispersion of induced amplitude and phase anisotropy of viscous solutions of dyes. J. Appl. Spectr. 65, 503–507 (1998).

[b38] Pikulik L. G., Chernyavskii V. A. & Grib A. F. Laser-radiation-induced optical anisotropy of rhodamine dye solutions. J. Appl. Spectr. 67, 791–795 (2000).

[b39] Kurnikova M. G., Balabai N., Waldeck D. H. & Coalson R. D. Rotational relaxation in polar solvents. Molecular dynamics study of solute-solvent interaction. J. Am. Chem. Soc. 120, 6121–6130 (1998).

[b40] Azzam R. M. A. Propagation of partially polarized light through anisotropic media with or without depolarization: A differential 4 × 4 matrix calculus. J. Opt. Soc. Am. 68, 1756–1767 (1978).

[b41] Bachelard N., Gigan S., Noblin X. & Sebbah P. Adaptive pumping for spectral control of random lasers. Nature Phys. 10, 426–431 (2014).

