# Supplementary material for: Circularly polarized laser emission induced in isotropic and achiral dye systems
Source: Sci Rep. 2016 Jun 28;6:28740. doi: 10.1038/srep28740 (PMC4923901; doi:10.1038/srep28740)
Supplement: Supplementary Information [file srep28740-s1.pdf]

<u>SUPPLEMENTARY INFORMATION</u>

to

Circularly polarized laser emission induced in isotropic and achiral dye systems

Luis Cerdán,*[1,2] Sara García-Moreno,[2] Angel Costela,[1] Inmaculada García-Moreno,[1] and Santiago de la Moya[2]

[1]*Instituto de Química Física "Rocasolano" (IQFR), Consejo Superior de Investigaciones Científicas (CSIC), C/ Serrano 119, 28006, Madrid (Spain)*

[2]*Facultad de Ciencias Químicas, Universidad Complutense de Madrid (UCM), Ciudad Universitaria S/N, 28040, Madrid (Spain)*

Polarimetry Formalism

The global polarization state of a given arbitrary beam can be described in terms of the Stokes parameters as [1]:

$$\begin{aligned} S_0 &= E_{out} \\ S_1 &= E_{out} DOP \cos 2\psi \cos 2\chi \\ S_2 &= E_{out} DOP \sin 2\psi \cos 2\chi \\ S_3 &= E_{out} DOP \sin 2\chi \end{aligned}$$

Where E_{out} is the beam energy, DOP is the degree of polarization, and ψ and χ are, respectively, the orientation and ellipticity of the polarization ellipse (Fig. S1a). Note here that $E_{out} \cdot DOP$, 2ψ and 2χ are the spherical coordinates of the three-dimensional vector of Cartesian coordinates (S_1, S_2, S_3) on the Poincaré sphere (Fig. S1b).

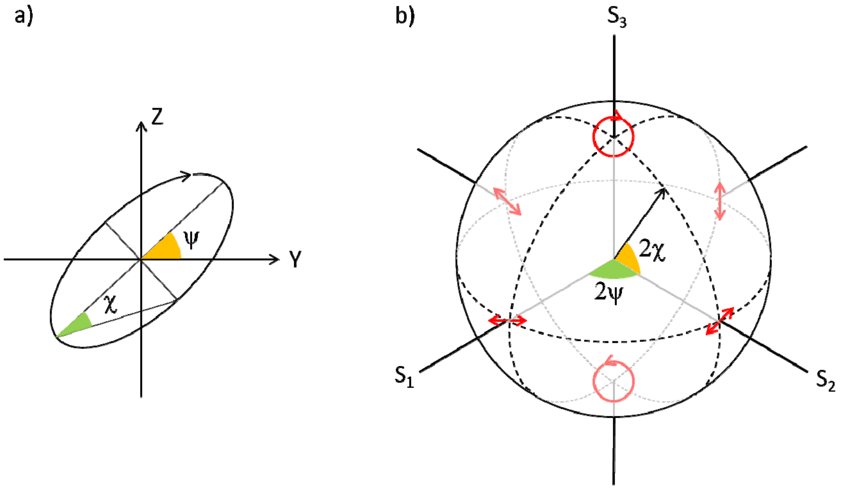

Fig. S1: a) Polarization ellipse and b) Poincaré sphere with representative polarization states.

These parameters can be alternatively expressed in terms of the Stokes parameters as:

$$
\begin{aligned}
&E_{out} = S_0 \\
&DOP = \frac{\sqrt{S_1^2 + S_2^2 + S_3^2}}{S_0}; DOCP = \frac{S_3}{S_0} \\
&2\psi = \arctan\frac{S_2}{S_1} \\
&2\chi = \arcsin\frac{S_3}{DOP \cdot E_{out}} = \arctan\frac{S_3}{\sqrt{S_1^2 + S_2^2}}
\end{aligned}
\tag{S1}
$$

For convenience, we have included in Eqs. (S1) the degree of circular polarization (*DOCP*), which will be used along the text. To measure the Stokes vector, we have used a home-made polarimeter with a rotating polarizer (or analyzer) and a static λ/4 retarder, a Fresnel Rhomb in our case (see Fig. 1), and applying the Mueller matrix formalism [2]. In this formalism, the Mueller matrix M is used to describe the polarization-altering characteristics of the given optical element (polarizer or λ/4 retarder). The arbitrary polarization state *S* of a beam crossing an optical element will be transformed into a new polarization state *S'*:

$$
S' = \begin{pmatrix} S_0' \\ S_1' \\ S_2' \\ S_3' \end{pmatrix} = MS = \begin{pmatrix} m_{00} & m_{01} & m_{02} & m_{03} \\ m_{10} & m_{11} & m_{12} & m_{13} \\ m_{20} & m_{21} & m_{22} & m_{23} \\ m_{30} & m_{31} & m_{32} & m_{33} \end{pmatrix} \begin{pmatrix} S_0 \\ S_1 \\ S_2 \\ S_3 \end{pmatrix}
$$

For the case of a linear polarizer with its transmission axis forming an angle θ above the horizontal, M is:

$$
M^{p} = \frac{1}{2} \begin{pmatrix} 1 & \cos 2\theta & \sin 2\theta & 0 \\ \cos 2\theta & \cos^2 2\theta & \sin 2\theta \cos 2\theta & 0 \\ \sin 2\theta & \sin 2\theta \cos 2\theta & \sin^2 2\theta & 0 \\ 0 & 0 & 0 & 0 \end{pmatrix}
$$

And for a λ/4 retarder with its fast axis forming an angle β above the horizontal, M is:

$$
M^{\lambda/4} = \begin{pmatrix} 1 & 0 & 0 & 0 \\ 0 & \cos^2 2\beta & \sin 2\beta \cos 2\beta & -\sin 2\beta \\ 0 & \sin 2\beta \cos 2\beta & \sin^2 2\beta & \cos 2\beta \\ 0 & \sin 2\beta & -\cos 2\beta & 0 \end{pmatrix}
$$

Finally, the Mueller matrix for an attenuator, which will eventually account for the transmission (*T*) in the optical elements, is given by:

$$M^{Att} = T^{Att}\begin{pmatrix}1 & 0 & 0 & 0\\ 0 & 1 & 0 & 0\\ 0 & 0 & 1 & 0\\ 0 & 0 & 0 & 1\end{pmatrix}$$

The only directly measurable Stokes parameter is S_0, as one only needs an energy meter to determine it. In order to retrieve the remaining parameters, one has to use a smart combination of polarizing elements that distil all the information on the initial Stokes parameters S_0 to S_3 into the exiting S_0'.

For example, the unknown polarization state S of a beam crossing a linear polarizer will be $S'=M^pS$, with S_0' equal to:

$$S_0' = E_{out}^{p}(\theta) = \frac{T^p}{2}\left[S_0 + S_1\cos 2\theta + S_2 \sin 2\theta\right] \tag{S2}$$

On the other hand, the unknown polarization state S of the same beam crossing first an arbitrarily oriented λ/4 retarder and later a linear polarizer will be $S''=M^pM^{\lambda/4}S$, with S_0'' equal to:

$$\begin{aligned} S_0'' = E_{out}^{p,\lambda/4}(\theta) = \frac{T^{p,\lambda/4}}{2}\Big[S_0 &+ \left(S_1\cos^2 2\beta + S_2 \sin 2\beta\cos 2\beta - S_3\sin 2\beta\right)\cos 2\theta \\ &+ \left(S_1 \sin 2\beta \cos 2\beta + S_2 \sin^2 2\beta + S_3\cos 2\beta\right)\sin 2\theta\Big] \end{aligned} \tag{S3}$$

In our experimental set-up β is set to ~π/2, so that Eq. (S3) approximates to:

$$S_0'' = E_{out}^{p,\lambda/4}(\theta) \approx \frac{T^{p,\lambda/4}}{2}\left[S_0 + S_1\cos 2\theta - S_3 \sin 2\theta\right] \tag{S4}$$

Hence, to retrieve S_0 to S_3 two sets of measurements must be performed. The first set corresponds to the energy of the beam as a function of θ after it has crossed the polarizer (*e.g.*, red data in Figs. 2 and S2). The second set corresponds to the energy of the beam as a function of θ after it has crossed first the λ/4 retarder at a given angle β (~π/2) and then the polarizer (*e.g.*, blue data in Figs. 2 and S2). A simultaneous fit of Eqs. (S2) and (S3) to the first and second sets of data, respectively, will provide S_0 to S_3 as fitting parameters. Once the Stokes parameters are known, the polarization state in terms of I_{out}, *DOP*, *DOCP*, ψ and χ, can be calculated using Eqs. (S1).

There are alternative parameters to account for the degree of circularization or ellipticity of a given light beam. For example, the works dealing with Circularly Polarized Luminescence (CPL) make use of the so-called dissymmetry factor g_{lum}, defined as:

$$g_{lum} = 2\frac{I_L - I_R}{I_L + I_R} \tag{S5}$$

Where I_L and I_R are the intensities of the left and right circularly polarized components of the emitted light. Therefore, g_{lum} values stand between −2 and +2 (completely right and left polarized emission, respectively). In

our current experimental set-up and methodology, $I_{R,L}$ would be the intensities of the beam after crossing the λ/4 retarder and later the polarizer with the latter at angles $\theta=\beta\pm\pi/4$, respectively, *i.e.*, $I_{R,L}=E_{out}^{p,\lambda/4}(\beta\pm\pi/4)$ in terms of Eq. (S3) and, accordingly:

$$g_{lum}=2\frac{E_{out}^{p,\lambda/4}(\beta-\pi/4)-E_{out}^{p,\lambda/4}(\beta+\pi/4)}{E_{out}^{p,\lambda/4}(\beta-\pi/4)+E_{out}^{p,\lambda/4}(\beta+\pi/4)}=-2\frac{S_3}{S_0} \qquad \text{(S6)}$$

Which is, in turn, minus twice the degree of circular polarization (*DOCP*) as defined in Eqs. (S1).

Polarimeter calibration

As small degrees of circular polarization in the total emitted laser intensity should be accurately detected, the sensitivity and reliability grade must be kept as best as possible. In order to avoid artefacts related to the non-perfect energy stability of the pump laser, and subsequent dye laser emission variation, each output energy data ($E_{out}(\theta)$) was averaged over 32 pulses (to smooth out pulse-to-pulse variations). In addition, the pump energy as detected by the trigger (signal) was as well averaged over 32 pulses and used to normalize the output intensity (to smooth out pump energy dwelling over extended periods of time). Hence, each set of measurements is obtained as follows: the analyzer angle θ is changed from 0 to 180º, and the energy is measured at each angle. A total of 10 set of measurements are acquired to obtain as better statistics as possible. Finally, as each set of measurements is fully independent, we can fit each set of measurement of the first kind (without λ/4 retarder) with each of the set of measurements of the second kind (with λ/4 retarder) as a partner. This renders a total of 100 fits with their corresponding errors for each pump energy, which increases the accuracy of the fitting procedure. Those 100 fitting parameters and errors are weighted averaged to retrieve a final value and error. With this stringent measurement protocol, the precision (errors cast by the fitting) for 2χ, which is the parameter we are interested at most, can be reduced down to 1 mrad. Nevertheless, the highest accuracy that we have achieved is of ±5 mrad due to unavoidable noise in the data. In other words, values of 2χ below 5 mrad are not accurately detected (they are overestimated). All the parameters and errors presented in this work are weighted averages.

In a first set of experiments, we address the calibration of the system to increase its sensitivity and reliability grade. As stated before, in our experimental set-up we set the Fresnel Rhomb with its fast axis at an angle $\beta\sim\pi/2$. But this value must be accurately determined, as small deviations in it lead to significant changes in 2χ (determined to be around 0.03 rad per degree of deviation). To determine β, we placed a polarizer with a fast/slow axis transmission ratio at the laser wavelength of 1:100000 between the dye laser cavity (containing PM567 5×10^{-4} M in ethyl acetate) and the polarimeter to ensure that the output beam was effectively linearly polarized ($S_3\sim0$). Fitting Eqs. (S2) and (S3) (with S_3 set to zero and leaving S_0, S_1, S_2, and β free) to the sets of data without and with λ/4 retarder, respectively, will provide β as a fitting parameter. After repeating the

experiment with five different polarizer orientations (see one of the orientations in Fig. S2) renders a weighted average $\beta \sim 93.76^{o} \pm 0.01^{o} = \pi/1.64$.

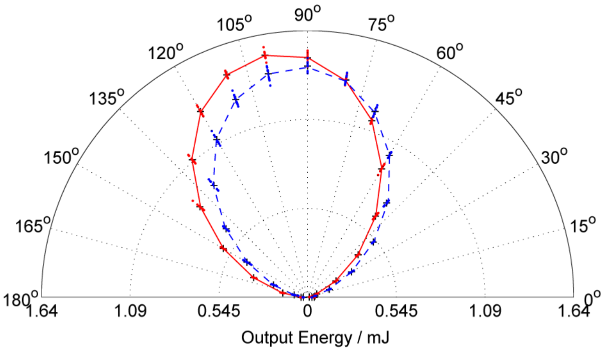

Fig. S2: Polarimeter calibration: Output energy as a function of the analyzer transmission axis angle θ without (red data) and with (blue data) inserting a $\lambda/4$ retarder in the beam path. Dots are experimental measurements and lines are best fits of Eqs. (S2) and (S3).

Variation of excited state population orientation distribution with pump polarization

The orientation dependant ground state population $\boldsymbol{\rho_g} = \rho_g^{\,0} \cdot \mathbf{e_d}(\theta,\phi)$, where $\rho_g^{\,0}$ is the doping concentration, and $\mathbf{e_d}(\theta,\phi)$ is the orientation distribution of absorption dipole moments unit vectors. Just before excitation, the ground state molecules (and therefore their dipole moments) are randomly and homogenously distributed. Hence, $\mathbf{e_d}(\theta,\phi)$ is constant and in spherical coordinates can be expressed as:

$$
\begin{aligned}
e_d^x &= \sin\theta\cos\phi \\
e_d^y &= \sin\theta\sin\phi \qquad \text{(S7)} \\
e_d^z &= \cos\theta
\end{aligned}
$$

During excitation, only the molecules whose absorption dipole moments unit vectors $\mathbf{e_d}$ are properly aligned with the electric field $\mathbf{E_p}$ of the pump radiation will be excited. The probability of being excited will be then proportional to $|\mathbf{E_p} \cdot \mathbf{e_d}|^2$, *i.e.*, it will be proportional to $\cos^2\vartheta$, where ϑ is the dihedral angle formed by the absorption dipole moment and the electric field vector. In an ensemble of randomly and homogenously distributed molecules, the orientation dependant excited state population $\boldsymbol{\rho_{ex}} = (\rho_{ex}^{\,x}, \rho_{ex}^{\,y}, \rho_{ex}^{\,z})$ in spherical coordinates will be then:

$$
\begin{aligned}
\rho_{ex}^x &= \rho_{ex}^0 \sin\theta\cos\phi \\
\rho_{ex}^y &= \rho_{ex}^0 \sin\theta\sin\phi \qquad \text{(S8)} \\
\rho_{ex}^z &= \rho_{ex}^0 \cos\theta
\end{aligned}
$$

Where $\rho_{ex}{}^{0}= \rho_{ex}{}^{0}(\theta,\phi)$ is now proportional to $|\mathbf{E_p}\cdot\mathbf{e_d}|^2$. The pump electric field for each pump polarization is given by:

$$\begin{aligned}
\overrightarrow{E_p} &= \left|E_p\right|(0,0,1);\ \text{Linear Vertical} \\
\overrightarrow{E_p} &= \left|E_p\right|\frac{1}{\sqrt{2}}(1,0,1);\ \text{Linear } 45^{\circ} \\
\overrightarrow{E_p} &= \left|E_p\right|(1,0,0);\ \text{Linear Horizontal} \\
\overrightarrow{E_p} &= \left|E_p\right|\frac{1}{\sqrt{2}}(1,0,i);\ \text{Circular}
\end{aligned} \tag{S9}$$

Hence, the orientation distribution of excited states $\rho_{ex}{}^{0}$ as a function of each pump polarization can be obtained from the square of the modulus of the scalar product of Eqs. (S7) and (S9). Removing the proportionality factors, $\rho_{ex}{}^{0}(\theta,\phi)$ is:

$$\begin{aligned}
\rho_{ex}^{0}(\theta,\phi) &\propto \cos^2\theta;\ \text{Linear Vertical} \\
\rho_{ex}^{0}(\theta,\phi) &\propto \frac{1}{2}\left(\sin\theta\cos\phi+\cos\theta\right)^2;\ \text{Linear } 45^{\circ} \\
\rho_{ex}^{0}(\theta,\phi) &\propto \sin^2\theta\cos^2\phi;\ \text{Linear Horizontal} \\
\rho_{ex}^{0}(\theta,\phi) &\propto \frac{1}{2}\left(\sin^2\theta\cos^2\phi+\cos^2\theta\right);\ \text{Circular}
\end{aligned} \tag{S10}$$

Figure 4a shows the qualitative representation of the density of excited state molecules $\boldsymbol{\rho_{ex}}=(\rho_{ex}{}^{x},\rho_{ex}{}^{y},\rho_{ex}{}^{z})$ upon excitation with different polarization states, represented as hue variations over a sphere.

Variation of refractive index with excited state population (Kramers-Kronig relations)

In the previous section it was demonstrated that the pump radiation excites preferentially molecular dipoles that are aligned with its electric field vector. Consequently, the molecules remaining in the ground state will present an orientation anisotropy that is conjugated with respect to that of the excited molecules. This, in turn, translates into an absorption orientation anisotropy. It is well-known that the real and imaginary parts of the medium susceptibility are connected by a causality relationship (Kramers-Kronig relations). In terms of quantities more useful for our purposes, absorption coefficient (α) and refractive index (n) are causally connected through the relation:

$$n(\omega)=1+\frac{c}{\pi}\wp\int_0^{\infty}\frac{\alpha(\omega)}{\Omega^2-\omega^2}d\Omega \tag{S11}$$

Where ω is the frequency in vacuum, c is the speed of light, and $\wp$ is the Cauchy principal value. The absorption coefficient is related to the ground state population density ρ_g as $\alpha(\omega)=\sigma_{abs}(\omega)(\rho_d- \rho_{ex})$,

where $\sigma_{abs}(\omega)$ is the absorption cross section, and ρ_d and ρ_{ex} are the total and excited state population densities. Introducing the expression of $\alpha(\omega)$ in Eq. (S11), it is straightforward to see that a variation of excited state population density $\Delta\rho_{ex}$ is followed by a change in refractive index of the excited medium $n_{ex}=n_0+\Delta n$, where n_0 is the refractive index of the passive medium and Δn is the variation in refractive index:

$$\Delta n(\omega) = -\Delta\rho_{ex} \frac{c}{\pi} \wp \int_0^{\infty} \frac{\sigma_{abs}(\omega)}{\Omega^2 - \omega^2} d\Omega = -cte\Delta\rho_{ex} \qquad \text{(S12)}$$

In other words, the higher the density of excited states becomes, the lower the refractive index is.

Supplementary figures

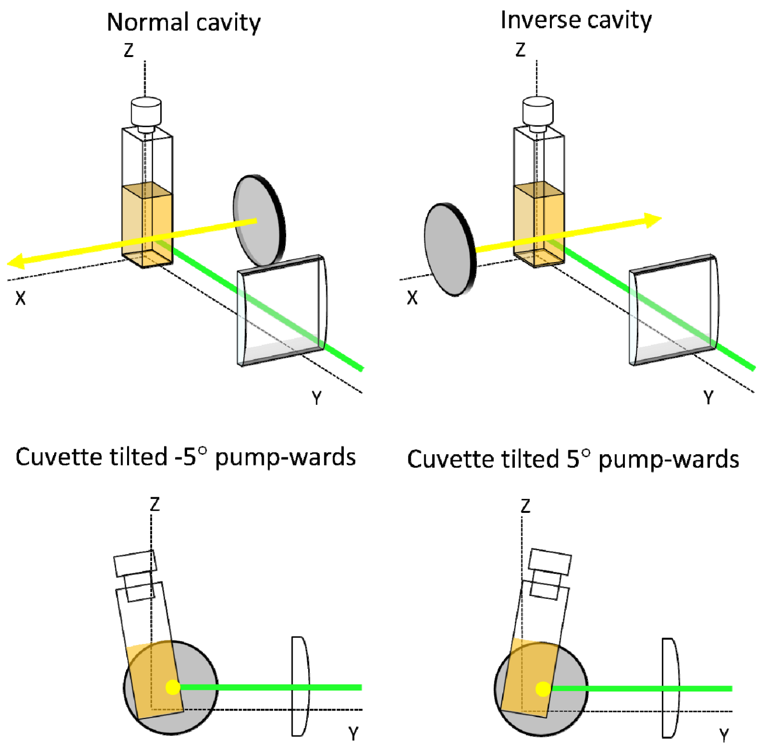

Fig. S4: Sketch of cavity configurations used in this work.

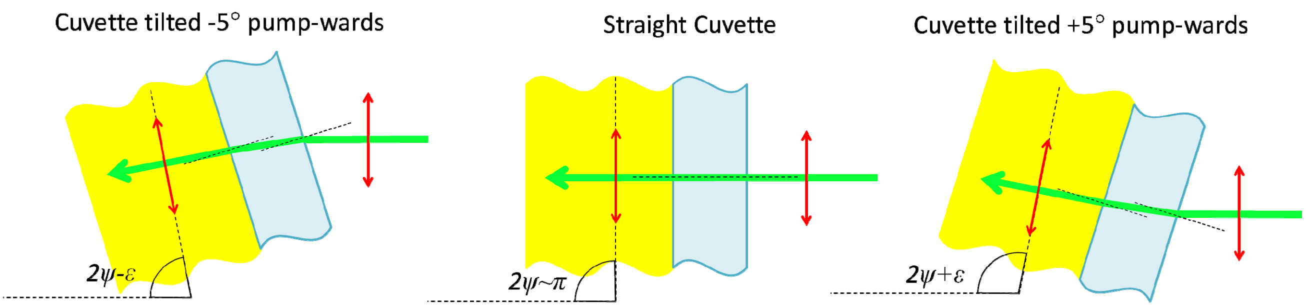

Fig. S5: Schematic representation of pump beam and electric field orientation as a function of cuvette tilting.

References

[1] M. Born and E. Wolf, *Principles of Optics*, 5th Ed. (Pergamon Press, 1975)

[2] R. A. Chipman, "Polarimetry," chapter 22 in *Handbook of Optics II*, 2nd Ed, M. Bass, editor in chief (McGraw-Hill, New York, 1995).
